# Surface thermal oxidation on titanium implants to enhance osteogenic activity and *in vivo* osseointegration

**DOI:** 10.1038/srep31769

**Published:** 2016-08-22

**Authors:** Guifang Wang, Jinhua Li, Kaige Lv, Wenjie Zhang, Xun Ding, Guangzheng Yang, Xuanyong Liu, Xinquan Jiang

**Affiliations:** 1 Department of Prosthodontics, Ninth People’s Hospital affiliated to Shanghai Jiao Tong University, School of Medicine, 639 Zhizaoju Road, Shanghai 200011, China; 2 Oral Bioengineering Lab, Shanghai Research Institute of Stomatology, Ninth People’s Hospital Affiliated to Shanghai Jiao Tong University, School of Medicine, Shanghai Key Laboratory of Stomatology, 639 Zhizaoju Road, Shanghai 200011, China; 3 State Key Laboratory of High Performance Ceramics and Superfine Microstructure, Shanghai Institute of Ceramics, Chinese Academy of Sciences, Shanghai 200050, China; 4 University of Chinese Academy of Sciences, Beijing 100049, China

## Abstract

Thermal oxidation, which serves as a low-cost, effective and relatively simple/facile method, was used to modify a micro-structured titanium surface in ambient atmosphere at 450 °C for different time periods to improve *in vitro* and *in vivo* bioactivity. The surface morphology, crystallinity of the surface layers, chemical composition and chemical states were evaluated by field-emission scanning electron microscopy (FESEM), X-ray diffraction (XRD), and X-ray photoelectron spectroscopy (XPS). Cell behaviours including cell adhesion, attachment, proliferation, and osteogenic differentiation were observed *in vitro* study. The ability of the titanium surface to promote osseointegration was evaluated in an *in vivo* animal model. Surface thermal oxidation on titanium implants maintained the microstructure and, thus, both slightly changed the nanoscale structure of titanium and enhanced the crystallinity of the titanium surface layer. Cells cultured on the three oxidized titanium surfaces grew well and exhibited better osteogenic activity than did the control samples. The *in vivo* bone-implant contact also showed enhanced osseointegration after several hours of oxidization. This heat-treated titanium enhanced the osteogenic differentiation activity of rBMMSCs and improved osseointegration *in vivo*, suggesting that surface thermal oxidation could potentially be used in clinical applications to improve bone-implant integration.

As a representative example of a metallic biomaterial, titanium-based materials are the optimal choice for dental and orthopaedic implants from the perspectives of both clinical application and scientific research^1^. However, the lack of surface bioactivity must be considered in regard to their practical application. To overcome their bio-inert nature, many efforts have been made to modify and functionalize the surface of titanium-based implants^2^. Additionally, a common view is that their inherent material characteristics, such as their surface charge, surface wettability, crystallinity, surface roughness, surface topography and surface chemistry, can affect both stem cell behaviour and *in vivo* osseointegration^3^,^4^,^5^. Inspired by the concept of bionics, many researchers have devoted themselves to constructing a bio-mimetic microenvironment for inducing stem cell differentiation into osteoblasts and enhancing new bone formation *in vivo*, including the construction of surface chemical and topographical cues^6^,^7^,^8^. With respect to simplicity and flexibility, a passive strategy to modulate the surface chemical and physical properties would be preferred in the design of titanium-based implants.

It is common knowledge that when titanium and its alloys are exposed to air or water, they spontaneously form a native titanium dioxide (TiO_2_) layer, which is the so-called passive condition of titanium^9^. The existence of this thin passive layer transforms them into biologically inert metals. This layer minimizes both metallic ion release from the implant to the surrounding tissues^10^ and adverse body reactions^11^, and it has been considered to be of great importance for successful osseointegration. The properties of this surface oxide layer can markedly influence the biocompatibility of titanium^12^,^13^. Thus, various surface oxidation methods, such as electrochemical treatment (anodic oxidation), chemical (acid and alkali) treatment, plasma spray deposition, sol-gel formation, ion implantation, and thermal oxidation, have been employed to develop a functional implant surface by changing the surface properties of the native passive layer to improve osseointegration^14^,^15^,^16^,^17^,^18^,^19^.

Among these, thermal oxidation is believed to be a relatively simple/facile and cost-effective method based on the formation of a thick and hard oxide on titanium surfaces. For oxidation treatment, temperature and time are the main parameters to obtain excellent TiO_2_ coating. Wide ranges of temperature and treatment time have been commonly utilized to modify the oxidation layer properties^20^,^21^,^22^,^23^. High temperatures (up to 800 °C) and longer thermal oxidation times cause oxide de-bonding. Oxide layers obtained at lower temperatures and shorter times are not sufficiently thick for use in specific tribological applications^24^,^25^. Therefore, a medium temperature oxidation for a suitable time would be preferred to obtain a layer of titanium oxide with good mechanical and chemical properties. In our previous studies, a thermal oxidation was performed at 450 °C in an air ambient atmosphere to form crystallization^26^,^27^. This has been proven to be an effective annealing process for improving the crystallization and biological properties and indicates that a temperature of 450 °C might be a suitable parameter for thermal oxidation modification. Thus, taking 450 °C as the oxidation temperature, we assumed that with a certain oxidation time, we could find a rather simple but effective way to improve the osteogenic activity of titanium.

In this study, acid-etched commercial pure titanium (Cp-Ti) was used as a control (denoted as TO-0) for comparison, and samples of acid-etched Cp-Ti treated with thermal oxidation at 450 °C in an air ambient atmosphere for 2, 4 and 6 hours were used as test groups (denoted as TO-2, TO-4 and TO-6). Both *in vitro* and *in vivo* studies were performed for osteogenic ability evaluation of these four titanium implants.

## Results

### Characterization of samples

**Figure 1:**
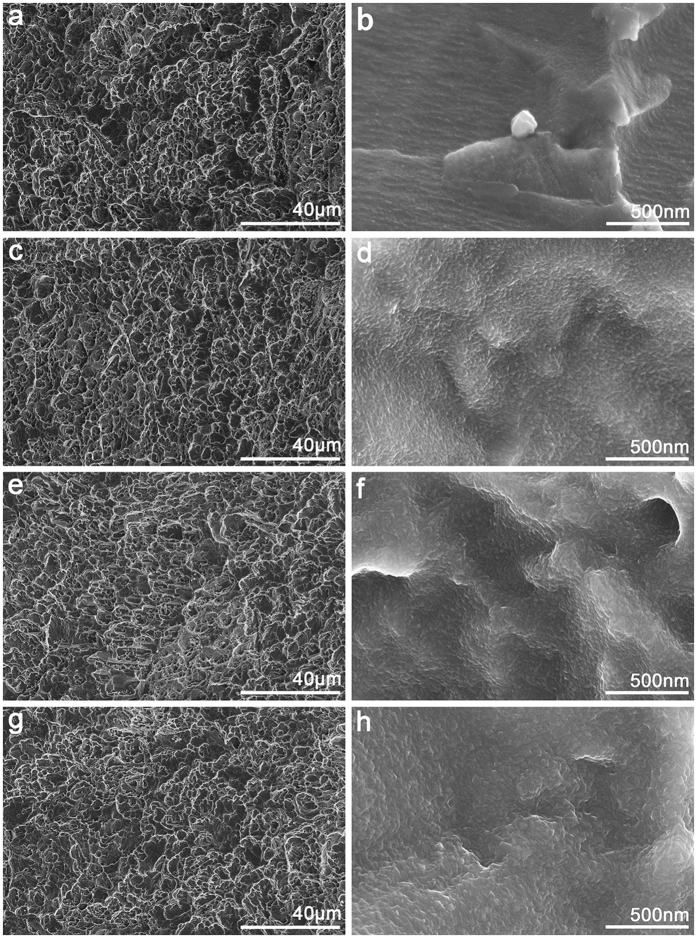
Surface morphology of four samples at different magnifications: (**a,b**) TO-0, (**c,d**) TO-2, (**e,f**) TO-4, (**g,h**) TO-6.

Figure 1 shows the surface topographies of Ti foils after undergoing acid etching and thermal oxidation treatments. The rough pits and crater-like holes of several micrometres or sub-micrometres were formed on the Ti surface by the strong acid etching with boiling oxalic acid solution (Fig. 1a). The high resolution images indicated that the ridges and valleys of the pits and holes were essentially smooth (Fig. 1b). With regard to samples TO-2, TO-4 and TO-6, the low-resolution images show that the surface structure did not change significantly. However, particle-like oxide appeared on the ridges and valleys of the pits and holes structure, and the carina size seemed to increase with prolonged thermal oxidation (Fig. 1c–h). Thus, these hierarchical surface structures were produced by a facile combination of acid etching and thermal oxidation treatments.

**Figure 2:**
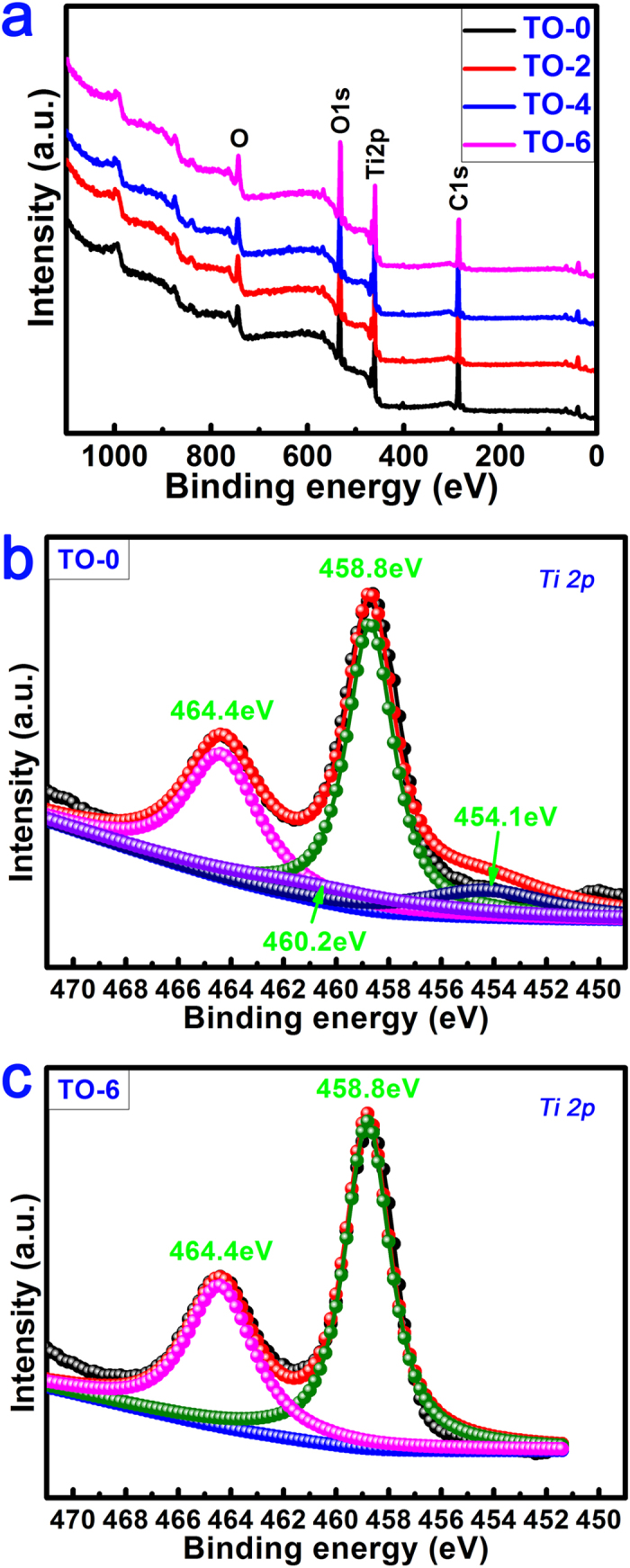
Surface chemical components and chemical states of different samples examined by XPS.

**Figure 3:**
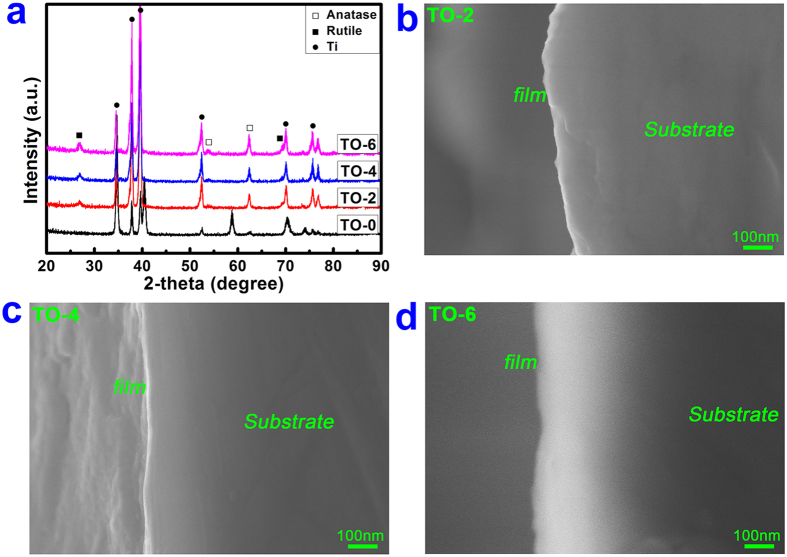
XRD pattern of the four specimens (**a**) and SEM images of cross-section of TO-2, TO-4 and TO-6 (**b–d**).

To investigate the surface chemical states of various samples, XPS analysis was carried out on each sample. Figure 2a shows the surface XPS full spectra of the samples TO-0, TO-2, TO-4 and TO-6. Table 1 gives the elemental compositions of various samples determined by XPS. A high level of oxygen content was detected on sample TO-0 due to the natural oxidation of pure titanium in the atmospheric environment^28^. After the thermal oxidation treatment for different periods of time, the oxygen content on the surface only exhibited a small increase according to the XPS data, which was mainly due to the sampling depth of the XPS analysis (~10 nm) of the outermost surface. The development of surface chemical compositions will be further analysed in the following. Figure 2b shows the high-resolution XPS spectrum of the Ti 2p peak for sample TO-0. The two peaks at 454.1 eV and 460.2 eV correspond to the metallic titanium^8^. Meanwhile, a strong doublet peak appeared at 458.8 eV and 464.4 eV with a broad shoulder. This peak should be attributed to the Ti 2p in Ti^4+^, which reveals that TiO_2_ is the predominant component of the surface oxidized layer. Likewise, for sample TO-6 (Fig. 2c), only the Ti 2p doublet peak (at 458.8 eV and 464.4 eV) ascribed toTiO_2_ was detected by XPS analysis. This result indicated the complete TiO_2_ overlay on the titanium surface after thermal oxidation treatment. To further analyse the development of the chemical composition of titanium surfaces, dispersive X-ray detector (EDX) analysis was also performed on samples TO-0, TO-2, TO-4 and TO-6.The EDX results are shown in Table 2. The oxygen percentage was 15.54% for the TO-0 group. After surface thermal oxidation treatment, oxygen significantly increased to 42.92%, 43.58% and 47.16% for the TO-2, TO-4 and TO-6 groups, respectively. Moreover, the oxygen content was not altered much between the TO-2 and TO-4 groups, both of which were slightly lower than the TO-6 group. To determine the crystallinity of this titanium dioxide layer on the Ti surface, XRD analysis was performed on various samples. In the XRD pattern of the acid-etched Ti, only the typical peaks of titanium were detected (Fig. 3a). After thermal oxidation for 2 h, typical diffraction peaks appeared at 2θ = 53.8° and 62.8° as indicators of the anatase TiO_2_ phase; the rutile TiO_2_ phase also emerged with two characteristic diffraction peaks at 2θ = 27.4° and 69.7°^29^. The diffraction peaks of both the anatase and rutile phases became increasingly strong with a prolonged thermal oxidation period. Figure 3b–d shows the SEM images of cross-sectional of samples TO-2, TO-4 and TO-6 examined by SEM from back scattered electron signal. From this figure, the thickness of oxide layer (TiO_2_) for samples TO-2 (Fig. 3b) and TO-4 (Fig. 3c) was about 30~50 nm; while for sample TO-6 (Fig. 3d), the thickness was appropriately 100~150 nm. This phenomenon was mainly due to the prolonged treatment duration of thermal oxidation of titanium substrate.

In this study, the relative weight percentage of rutile in TO-2, TO-4 and TO-6 with a mixture of anatase and rutile can be roughly estimated by use of the following formula^26^.


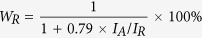


where *W*_*R*_ represents the relative weight percentage of rutile; *I*_*A*_ represents the X-ray integrated intensities of the (101) diffraction of anatase and *I*_*R*_ is the integrated intensities of the (110) diffraction of rutile. With prolonged treatment time, the W_R_ for the TiO_2_ layer increased from 58.7% in the TO-2 group to 67.2% in the TO-6 group. However, there was a slight decrease to 57.6% in the TO-4 group.

### Surface wettability

**Figure 4:**
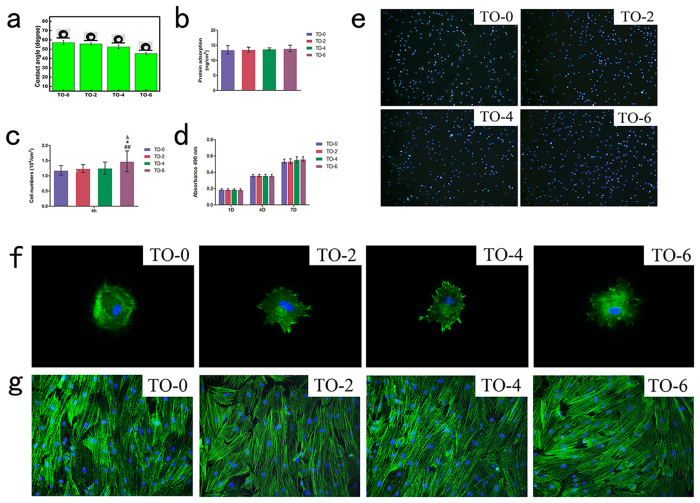
Surface biocompatibility. (**a**) Optical images of water contact angles, (**b**) Adsorption of proteins on different titanium surfaces. (**c**) Statistical results for adhesive cell numbers. (**d**) MTT assay for cell metabolism on titanium substrates. (**e**) Cell nuclei stained with DAPI at 4 hours after seeding at a 100-foldmagnification. (**f**) Actin cytoskeletons were labelled to observe cell attachment at 24 hours after seeding at 400-foldmagnification. (**g**) Actin cytoskeletons were labelled to observe cell morphology 7 days after cell-seeding on these four titanium surfaces.

The water contact angles of the TO-0, TO-2, TO-4 and TO-6 samples are shown in Fig. 4a. From these data, it can be seen that the thermally oxidized titanium surfaces became more hydrophilic than the control, TO-0, especially for the TO-6 sample.

### Protein adsorption

Figure 4b shows the adsorption concentration of protein on titanium surfaces after 4 hours of incubation. No significant differences were observed among those four groups.

### The effects of the titanium surface on BMMSCs *in vitro*

#### Cell adhesion, morphology, and growth of seeded rBMMSCs

**Figure 5:**
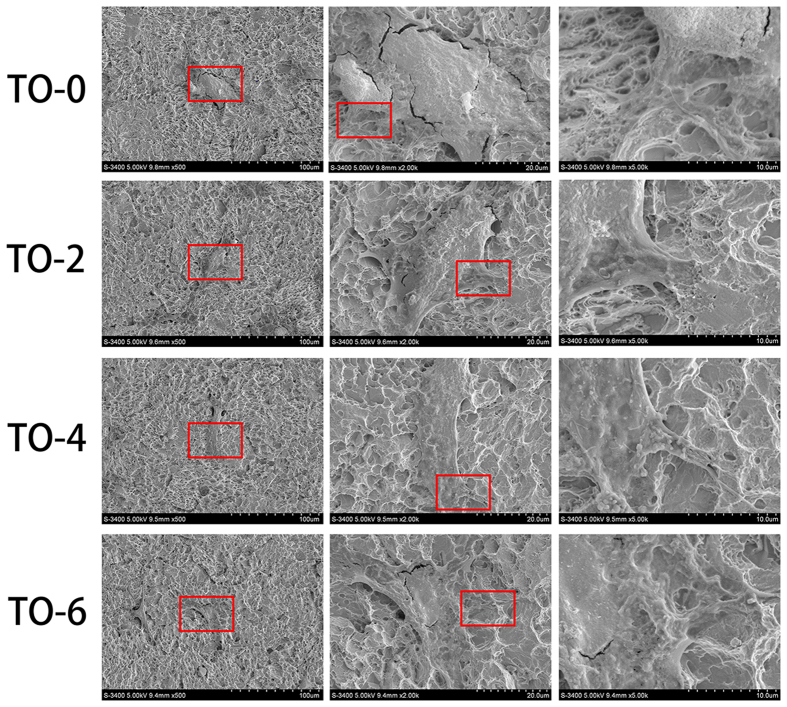
SEM observation of cell morphology on titanium surfaces after 2 days of seeding.

After 4 hours of culture, the cell adhesion quantitative results (Fig. 4c,e) exhibited that there were no significant differences among the TO-0, TO-2 and TO-4 groups, whereas there were more adhered cells on the TO-6 titanium surface compared to the other three samples. Cells attached to the control titanium surface spread well, whereas the thermally oxidized titanium surfaces exhibited better attachment (Fig. 4f). After 2 days of incubation, cells spread well on the thermally oxidized specimens (Fig. 5). After 7 days of culture, the cells had grown well and reached complete confluence in these four groups (Fig. 4g).

### Cell proliferation

MTT cell metabolic activity assay (Fig. 4d) was carried out to investigate cell proliferation on the titanium surfaces during the first 7 days of culture. No statistically significant differences were observed among the four samples on days 1, 4 or 7. The total cell metabolic activity on the control surfaces was not significantly greater or smaller than those on the thermal-oxidation-treated titanium surfaces.

### Cell differentiation

**Figure 6:**
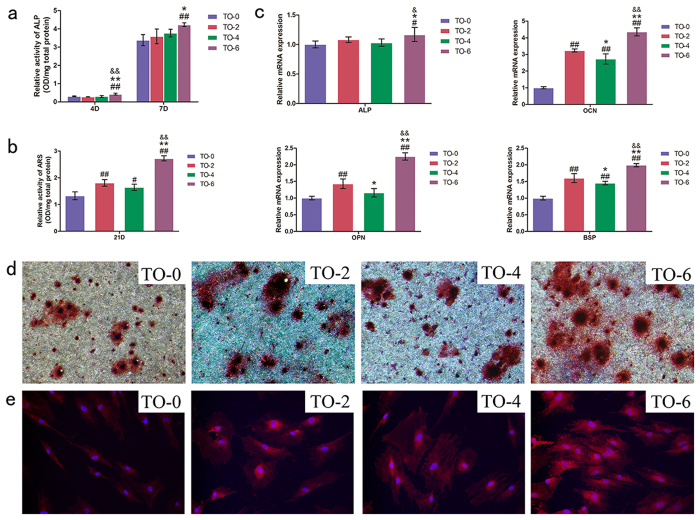
Osteogenic differentiation. Alkaline phosphatase (**a**) and matrix mineralization (**b**) semi-quantitative assay; (**c**) Expression of osteogenic-related differentiation genes (ALP, OCN, OPN and BSP) were measured by real-time PCR. (**d**) Alizarin Red S staining at 10-fold magnification. (**e**) Expression of OCN was detected by immunofluorescent staining after 14 days culture.

The alkaline phosphatase (ALP) semi-quantification results are shown in Fig. 6a. After 4 and 7 days of incubation, there was no significant enhancement detected in the TO-2 or TO-4 groups compared to the control samples. The secretion of ALP was significantly improved in the TO-6 group compared to the other three groups. After 21 days of culture, as measured using Alizarin red S staining, optical images were obtained and are shown in Fig. 6d. All of the thermally oxidized titanium surfaces were stained stronger than the non-thermally treated titanium surfaces for Alizarin Red S. In particular, the staining colour showed best in the TO-6 group. As shown in Fig. 6b, matrix mineralization was increased in those three thermal-oxidation-treated titanium surfaces. The TO-6 group exhibited the best matrix mineralization among those four titanium surfaces. The TO-2 group exhibited better matrix mineralization than the TO-4 group, although the difference was not significance. The relative mRNA expressions of osteogenesis-related genes, including ALP, osteocalcin (OCN), osteopontin (OPN), and bone sialoprotein (BSP) were illustrated in Fig. 6c. No significant differences were detected in the expression of ALP mRNA among the TO-0, TO-2 and TO-4 groups. The expression of ALP mRNA of the TO-6 group was significantly higher than that of other groups. The expressions of OCN, OPN, and BSP were up-regulated on the thermal-oxidation-treated titanium samples. Cells incubated on the TO-6 group exhibited the highest expression of these relatively late-stage markers. Cells cultured on the TO-2 group showed a better expression than did the TO-4 titanium surfaces. As shown in Fig. 6e, cells seeded on the TO-6 group expressed the highest level of OCN compared to the other three groups. The cells seeded on the TO-2 and TO-4 groups expressed a higher level compared to the control titanium surface. It was difficult to determine which group exhibited better OCN expression between the TO-2 and TO-4 groups.

### *In vivo* osseointegration

**Figure 7:**
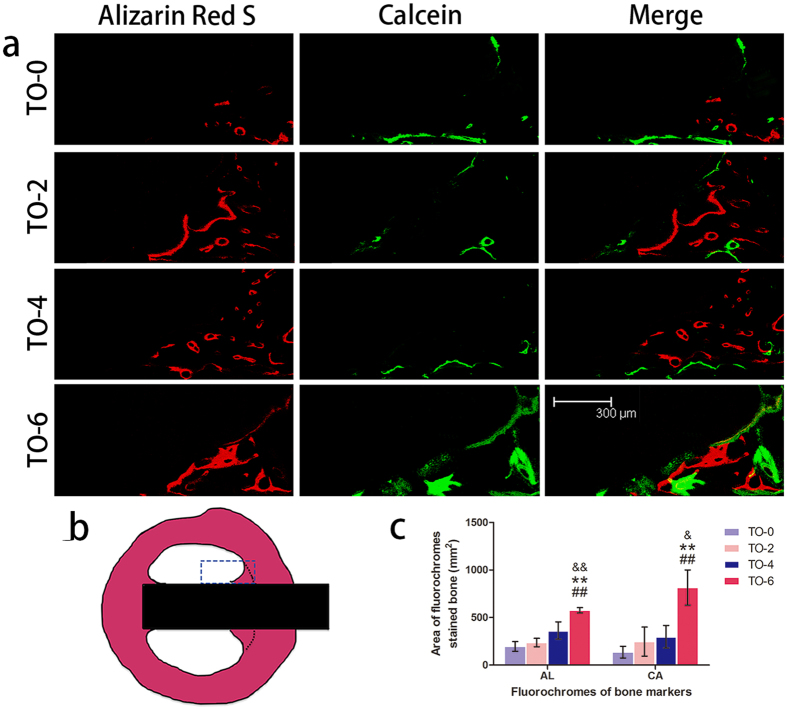
Sequential fluorescent labelling observations. (**a**) Red and green represent labelling by Alizarin Red S (AL) and Calcein (CA), respectively (bar = 300 um). (**b**) The blue rectangle region was selected to evaluate the new bone rate. (**c**) The area of the two fluorochromes stained bone.

**Figure 8:**
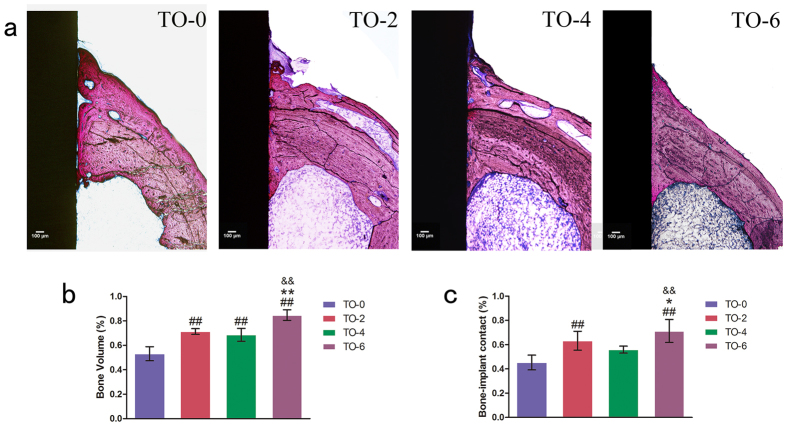
Histological observations and histomorphometric measurements. (**a**) Undecalcified sections are stained with Van Gieson’s picro fuchsin. The results of BV/TV (**b**) and BIC (**c**) from the histomorphometric measurements. (Notes: ^#^P < 0.05, ^##^P < 0.01 versus TO-0 group; *P < 0.05, **P < 0.01 versus TO-2 group; ^&^P < 0.05, ^&&^P < 0.01 versus TO-4 group).

Two types of fluorochromes at 6 and 9 weeks time point were used to assess newly formed bone area. As illustrated in Fig. 7, thermal oxidation treated titanium implant, TO-6, promoted the area of newly formed bone, compared to the other three groups. At different time points after implantation, the Alizarin Red S labelling area and calcein labelling area for the TO-6 titanium implant were significantly higher than those of the other three groups. The differences among the latter three groups were not significant. As illustrated in Fig. 8, the thermal-oxidation-treated implants showed significantly more BV/TV compared to the TO-0 implant. The TO-6 group exhibited the highest BV/TV. There was a significantly higher BV/TV in the TO-2 group than in the TO-4 group. The TO-2, TO-4 and TO-6 groups had significantly more bone contact than did the TO-0 group. The TO-4 group had less bone contact compared to the TO-2 group, but it was not a statistically significant difference. The TO-6 group had the most bone contact among these four groups.

## Discussion

Titanium-based materials are the ultimate choice for orthopaedic implants. To meet all of the clinical needs, their biocompatibility has been explored for possible improvement. The properties of the surface oxide is an important aspect in influencing the biocompatibility of titanium^30^. Titanium oxide exists in three different crystal lattices, including anatase, rutile and brookite. In our study, the surface of the acid-etched titanium contains a thin amorphous layer that is naturally oxidized upon exposure to the atmosphere, with a typical thickness of 3–7 nm and a main component of stable TiO_2_. After thermal oxidation treatment, the thickness of oxide layer was significantly increased. The crystallinity of this surface TiO_2_ layer was significantly changed and was composed of anatase and rutile crystal phases. According to the analysed results, the longest treatment time of 6 hours led to the highest W_R_ and the best osteogenic ability both *in vitro* and *in vivo*. It has been reported that anatase film can attract calcium and phosphate ions from the physiological environment to form an apatite coating. In contrast, the rutile film on titanium was associated not only with basic hydroxyl groups on the surface but also acidic hydroxyl groups and surface energy. The mixture of rutile and anatase might be responsible for enhancing the osteogenic properties of this biomaterial. The existence of both anatase and rutile could help improve the osteogenic activity of titanium.

In addition to surface oxide crystallinity, surface wettability is believed to be an important factor in the bioactivity of the titanium surface. The contact angles on the thermal-oxidation-treated Ti surfaces were significantly lower than on the control plate, and a prolonged heat-treatment time gradually decreased the contact angle. Surface morphology and chemical components are the two dominant factors affecting material wettability^31^. The observed enhancement in surface wettability might be partially affected by changes in titanium oxide crystallinity. It might more likely be attributable to the gradually changing titanium nano-scaled surfaces. After etching with oxalic acid solution, the titanium surface showed a rather homogeneous micro-scaled surface. Following oxidation treatment for 2, 4 and 6 hours, the micro-scaled surfaces on different samples remained the same as the non-heat treated titanium surfaces. However, in the high-resolution images, particle-like oxide appeared on the ridges and valleys of the pits and holes structure, which was rather smooth in the control group. With a prolonged oxidation time, the particle-like oxide grew sturdier from approximately an average of 20 nm in the TO-2 group to an average of 60 nm in the TO-6 group, which has been shown to influence surface wettability^32^.

Along with the changed wettability, in our study, there were no significant differences in protein adsorption observed among the specimens. The initial cell adhesion ability of titanium surfaces was not improved until the thermal oxidation treatment was prolonged to 6 hours. Though the initial cell attachment observed by cytoskeleton staining showed that cells cultured on oxidized titanium surfaces seemed to spread out better than on the control group, there was no obvious difference in cell morphology among the non-oxidized titanium surface and the oxidized titanium surfaces after a short period of time, as observed in both fluorescence staining and SEM. This phenomenon suggested that the minor difference in surface wettability in our study did not have the ability to affect protein adsorption or cell attachment.

The interactions among titanium, titanium oxide, proteins and cells were complex. When titanium surfaces were treated with thermal oxidation for 2 and 4 hours, the titanium surface topography, titanium oxide crystallinity, oxide layer thickness and surface wettability were altered. These changes alone or together played a positive role in promoting the osteogenic activity of the thermal-oxidation-treated titanium surfaces. However, these changed titanium surface characteristics did not have the ability to influence titanium surface protein adsorption or the cell response, including initial cell attachment, cell adhesion, or cell morphology. Compared to control group, TO-2 and TO-4 did not significantly promote the new bone formation *in vivo* either. After 6 hours of thermal oxidation treatment, the nano-scaled titanium surface became sturdier, the rutile ratio was increased and the oxide layer thickness increased, followed by promoted cell adhesion and enhanced osteogenic activity *in vitro*. Despite the lack of a significant difference in these cell functions, including cell attachment, cell adhesion and cell morphology, both the osteogenic activity *in vitro* and BIC *in vivo* were improved. Cells cultured on the three oxidized titanium surfaces grew well and exhibited better osteogenic activity than on the control samples. The fluorochrome labelling results at early stages of implantation could reflect the promoted new bone formation and mineralization in the TO-6 group. With treatment times of 2 and 4 hours, there were no significant differences compared to the control group found in bone regeneration or mineralization assay, which was probably caused by the weak enhancement in bone regeneration and mineralization after a shorter treatment time of 2 or 4 hours compared to a 6-hour treatment. The *in vivo* bone implant contact and bone volume ratio results also showed enhanced osseointegration after several hours of oxidization. With 6 hours of treatment, the TO-6 titanium implant exhibited the best osseointegration *in vivo.* This enhanced osteogenic activity can probably be attributed to the complex interactions among altered nano-scale titanium surface topography, changed titanium oxide crystallinity, increased oxide layer thickness and the enhanced wettability. However, it is difficult to determine how these elements interact or which element plays the most important role in this cell function enhancement. It is interesting to note that the TO-4 group exhibited less up-regulated osteogenic activity *in vitro* and worse osseointegration *in vivo*, though non-significantly. In agreement with the cell response tendency *in vitro* and *in vivo*, it was surprising to find that the W_R_ for the TiO_2_ layer of the TO-6 group was the highest, whereas that of the TO-4 group was lower than that of the TO-2 group. A previous study demonstrated that with the addition of rutile TiO_2_ on the titanium surface, its ALP activity was increased^33^. According to our results, we hypothesize that the relative weight percentage of rutile for the TiO_2_ layer might be positively correlated with the osteogenic activity and osseointegration of the titanium surface.

With regard to the enhancement effect of thermally oxidized titanium surface on the osteogenic activity, a plausible mechanism was proposed from the following two points. On the one hand, the construction of surface hierarchical structure may endow titanium surface with favorable osteogenic activity^34^. On the other hand, the metallic titanium surface is intrinsically bioinert although the outermost surface contains a thin layer of nanocrystalline TiO_2_ with thickness of 3~7 nm^2^. After thermal oxidation, the thickness of oxide layer on TO-2 and TO-4 increased to 30~50 nm. And for TO-6, the thickness ascended to 100~150 nm. The increase in thickness of the oxide layer contributed to the enhanced osteogenic activity. Meanwhile, the difference in oxide layer thickness between TO-6 and TO-2 (or TO-4) may also account for the distinct osteogenic activity of the thermally oxidized titanium surface.

This oxidation method requires neither a high reaction temperature nor complicated reaction processes. It can be used to produce micro- and nano-scale modified titanium implants with relatively long reaction times at rather low temperatures. It enhanced the osteogenic differentiation activity of rBMMSCs and improved osseointegration *in vivo*, thus suggesting that surface thermal oxidation could potentially be used in clinical applications to improve bone-implant integration.

## Materials and Methods

### Animals

The animals were acquired from the Ninth People’s Hospital Animal Center (Shanghai, China).The experimental protocol was approved by the Animal Care and Experiment Committee of the Ninth People’s Hospital, and the methods were performed in accordance with the approved guidelines.

### Specimen preparation

Firstly, commercial pure titanium (Cp-Ti, Grade 1) foils with dimensions of 1.0 cm × 1.0 cm × 0.1 cm or 2.0 cm × 2.0 cm × 0.1 cm were ultrasonically cleaned in ethanol. Then, they were deionized with water several times and pickled in 5 wt % oxalic acid solution at 100 °C for 2 hours. Titanium surface was etched and homogeneous surface microstructure was obtained. Subsequently, the Ti foils were ultrasonically cleaned in deionized water and dried in ambient atmosphere for further use. After acid etching, thermal oxidation treatment was conducted on the Ti foils. In detail, the furnace was first heated up to 450 °C, with a heating rate of 5 °C/min. The temperature was kept at 450 °C for periods of 2 h, 4 h or 6 h. Subsequently, the furnace was naturally cooled to room temperature. Meanwhile, pure medical titanium rods (Grade 1) with an external diameter of 0.2 cm and a length of 0.7 cm were used in the animal experiments. The acid-etched Ti was used as the control group (denoted as TO-0). The thermally oxidized Ti was used as the experimental group (denoted as TO-2, TO-4 and TO-6).

### Specimen characterization

A field emission scanning electron microscope (FESEM; Magellan 400, FEI, Hillsboro, Oregon, USA) equipped with an energy dispersive X-ray detector (EDX) was used to characterize the surface morphologies of the four titanium surfaces. The crystallinity of the surface layers was investigated using an X-ray diffractometer (XRD; D/Max, Rigaku, Tokyo, Japan) fitted with a Cu Kα (λ = 1.541 Å) source at 40 kV and 100 mA in the range of 2θ = 10–90° with a step size of 0.02°. Phase identification was performed using the standard JCPDS database. In the X-ray diffraction experiments, the glancing angle of the incident beam against the sample surface was fixed at 1°. The chemical compositions and chemical states of the Ti surfaces were determined by X-ray photoelectron spectroscopy (XPS; PHI 5802, Physical Electronics Inc, Eden Prairie, MN) with an Mg Kα (1253.6 eV) source.

### Contact angle measurements

To assess surface wettability of the four samples, contact angles were measured at room temperature with atmospheric relative humidity of 30% (Automatic Contact Angle Meter Model SL200B, Solon information technology Co., Ltd, China). Five drops of contact angle measurements were performed for each sample. The experiment was repeated twice.

### Protein adsorption

Dulbecco’s modified Eagle’s medium (DMEM) containing 10% foetal bovine serum (FBS) was used to examine the protein adsorption of different titanium specimens. Different titanium surfaces were immersed in 1 mL of DMEM for 4 hours at 37 °C. After washing thrice with 1 mL phosphate balanced solution (PBS), 500 μL of 1% sodium dodecyl sulfate (SDS) solution was added to each well on these substrates. Then, the substrates were shaken for 1 hour to detach proteins. The concentrations of protein collected in SDS solutions were measured using a Micro BCA protein assay kit (Thermo Fisher Scientific, Waltham, MA, USA).

### *In vitro* biocompatibility analysis

#### Cell culture

rBMMSCs were isolated and cultured from 4-week-old male SD rats according to previously published procedures^35^. The bone marrow was rinsed using DMEM with 10% FBS from rat femurs after both ends were cut off at the epiphysis. Cells were cultured with an atmosphere of 5% CO_2_ at 37 °C. After 48 hours of incubation, the medium was changed. Cells at passage 2–3 were used in the following experiments.

#### Cell adhesion and morphology

rBMMSCs were cultured on different titanium surfaces in 24-well plates at a density of 5.0 × 10^4^ and 2.0 × 10^4^ cells per ml for cell adhesion and morphology, respectively. At day 4 and 7, the remaining cells were fixed in 4% paraformaldehyde, then, treated with 0.2% Triton X-100 in PBS for 30 min. Subsequently, specimens were incubated with 100 nM FITC-phalloidin (Cytoskeleton Inc, Acoma St. Denver, USA) for 60 min, stained with 1 μM 4′, 6-diamidino-2-phenylindole (DAPI, Thermo Fisher Scientific, Waltham, MA, USA) for 15 min. Finally, they were mounted on glass slides and observed^36^. The cell numbers in five random fields of each sample at 4 hours were counted under a fluorescence microscope for a cell adhesion ability assay^28^. After 2 days of culture, cells were fixed in 2.5% glutaraldehyde overnight at 4 °C. Then, they were dehydrated by increasing concentration of ethanol. Finally, the samples were dried by hexamethyldisilazane, sputter-coated with gold and examined by SEM^37^.

#### Cell proliferation

For the cell proliferation assay^38^, cells were cultured on the four specimens for 1, 4 and 7 days. At each time point, 1 mL DMEM supplemented with 100 μL of 5 mg/mL MTT solution was supplemented to each targeted well. After 4 hours of culture, the medium were replaced with 300 μL of dimethyl sulfoxide (DMSO) and vibrated for 15 min. Finally, the absorbance was measured at 490 nm by ELX Ultra Microplate Reader (BioTek, Winooski, VT, USA).

#### Cell differentiation

The cells were cultured on the substrates for 4 and 7 days. A semi-quantitative analysis of ALP was carried out according to previously described procedures^39^. After incubation with p-nitrophenyl phosphate (Sigma-Aldrich, St Louis, MO, USA), optical density (OD) values for absorbance at 405 nm was measured to determine ALP activity. After 21 days of culture, specimens were treated with 4% paraformaldehyde for fixation, washed with distilled water and stained with 1% Alizarin Red S for mineralization examination. They were washed several times with distilled water and observed. For the quantification analysis, the stained samples were desorbed by use of 10% cetylpyridinium chloride (Sigma). Absorbance values at 590 nm were recorded. Intracellular total protein content was determined by use of the microBCA protein assay kit. ALP and calcium deposition quantity analyses results were normalized to the total protein content^40^.

The expressions of osteogenesis-related genes ALP, OCN, OPN and BSP were evaluated using real-time polymerase chain reaction (Real-time PCR). After cells were cultured on different substrates for 14 days, total RNA was isolated using the TRIzol reagent. Complementary DNA (cDNA) was synthesized using the PrimeScript^TM^ RT reagent kit (TaKaRa, Bio Inc, Otsu, Japan). Primer sequences for the selected genes were the same as those described in previous studies^41^,^42^. Expressions of these genes were quantified by use of Real-time PCR with SYBR Premix Ex Taq II (TaKaRa, Bio Inc, Otsu, Japan). The relative expression levels for each gene of interest were normalized to that of the housekeeping gene GAPDH. Immunofluorescent staining was carried out to detect the expression of osteocalcin protein. Briefly, cells were fixed in 4% paraformaldehyde after they were cultured on each specimen for 14 days. After washed with PBS for three times, cells were permeabilized with 0.2% Triton X-100 in PBS, and then blocked with 10% goat serum for 1 hour. Specimens were incubated in rat specific primary antibodies against OCN (Abcam Inc, Cambridge, MA, USA) overnight at 4 °C. Then, they were incubated with DyLight 549 conjugated anti-mouse IgG antibody (Jackson ImmunoResearch Inc, West Grove, PA, USA) for another 1 h at room temperature. Cellular nuclei were counterstained with DAPI. All specimens were mounted on glass slides and observed.

### *In vivo* osseointegration evaluation

#### Surgical procedures

Four male white New Zealand rabbits with an average weight of 2.5 kg were used for the implantation of the following 4 groups of implants: (A) oxalic acid-etched titanium rods (TO-0 group, n = 8), (B) oxalic acid-etched titanium followed with thermal oxidation treatment for 2 hours (TO-2 group, n = 8), (C) oxalic acid-etched titanium followed with thermal oxidation treatment for 4 hours (TO-4 group, n = 8), and (D) oxalic acid-etched titanium followed with thermal oxidation treatment for 6 hours (TO-6 group, n = 8). The surgery was conducted according to previously published procedures^36^. New Zealand rabbits were anesthetized with the intramuscular injection of ketamine at a dose of 40 mg/kg. A lateral longitudinal skin incision was made to expose the mid-shaft of the femur. Four surgical sites were prepared on the shaft of each femur. Four types of implants were randomly placed into each rabbit, four per femur.

#### Sequential fluorescent labelling

A polychrome sequential fluorescent labelling method was carried out to assess newly formed bone area. At 6 and 9 weeks post-operation, the animals were administered intraperitoneally with 30 mg/kg Alizarin Red S and 20 mg/kg Calcein (Sigma), respectively.

#### Histomorphometric observation

At 12 weeks post-operation, all animals were sacrificed. Bilateral femurs containing the implants were harvested and fixed in 10% buffered formaldehyde. After trimmed into small ones, specimens were dehydrated in an ascending series of alcohols and embedded in poly-methylmethacrylate (PMMA). They were then cut into 150-μm-thick sections using a saw microtome (Leica microsystems, Hamburg, Germany), ground and polished to a final thickness of about 40 μm. The sections were stained with van Gieson’s picro fuchsin and observed. To evaluate the effects of osseointegration^43^, the percentage of bone implant contact (BIC) and bone volume ratio (BV/TV) within the 100 μm layer adjacent to the titanium implant surface was calculated with Image-Pro Plus^TM^ software (Media Cybernetics, Silver Spring, MD, USA).

### Statistical analysis

Statistically significant differences (P < 0.05 and P < 0.01) among the various groups were measured by one-way ANOVA and SNK post hoc analysis. All statistical analyses were carried out using the SAS 8.2 statistical software package (SAS Institute, Cary, NC, USA). Results are presented as the mean ± standard deviation.

## Additional Information

**How to cite this article**: Wang, G. *et al.* Surface thermal oxidation on titanium implants to enhance osteogenic activity and *in vivo* osseointegration. *Sci. Rep.* **6**, 31769; doi: 10.1038/srep31769 (2016).

## Figures and Tables

**Table 1:** Percent contents of C, O and Ti elements for various samples determined by XPS.

sample	Elemental content (at.%)		
	C	O	Ti
TO-0	58.03	31.26	10.71
TO-2	55.60	32.48	11.92
TO-4	53.84	33.93	12.23
TO-6	51.03	36.15	12.82

**Table 2:** Percent contents of C, O and Ti elements for various samples detected by EDX.

sample	Elemental content (at.%, Mean ± SD)		
	C	O	Ti
TO-0	5.16 ± 0.29	15.54 ± 0.86	79.30 ± 0.98
TO-2	2.89 ± 0.08	42.92 ± 0.70	54.18 ± 0.77
TO-4	2.60 ± 0.16	43.58 ± 0.53	53.82 ± 0.69
TO-6	2.65 ± 0.37	47.16 ± 0.49	50.18 ± 0.60
